# Chemogenetic Recruitment of Specific Interneurons Suppresses Seizure Activity

**DOI:** 10.3389/fncel.2018.00293

**Published:** 2018-09-05

**Authors:** Alexandru Cǎlin, Mihai Stancu, Ana-Maria Zagrean, John G. R. Jefferys, Andrei S. Ilie, Colin J. Akerman

**Affiliations:** ^1^Department of Pharmacology, University of Oxford, Oxford, United Kingdom; ^2^Division of Physiology and Neuroscience, Department of Functional Sciences, Carol Davila University of Medicine and Pharmacy, Bucharest, Romania

**Keywords:** epilepsy, seizure, parvalbumin, somatostatin, vasoactive intestinal peptide, DREADDs, hM_3_D_q_, CNO

## Abstract

Current anti-epileptic medications that boost synaptic inhibition are effective in reducing several types of epileptic seizure activity. Nevertheless, these drugs can generate significant side-effects and even paradoxical responses due to the broad nature of their action. Recently developed chemogenetic techniques provide the opportunity to pharmacologically recruit endogenous inhibitory mechanisms in a selective and circuit-specific manner. Here, we use chemogenetics to assess the potential of suppressing epileptiform activity by enhancing the synaptic output from three major interneuron populations in the rodent hippocampus: parvalbumin (PV), somatostatin (SST), and vasoactive intestinal peptide (VIP) expressing interneurons. To target different neuronal populations, promoter-specific cre-recombinase mice were combined with viral-mediated delivery of chemogenetic constructs. Targeted electrophysiological recordings were then conducted in an *in vitro* model of chronic, drug-resistant epilepsy. In addition, behavioral video-scoring was performed in an *in vivo* model of acutely triggered seizure activity. Pre-synaptic and post-synaptic whole cell recordings in brain slices revealed that each of the three interneuron types increase their firing rate and synaptic output following chemogenetic activation. However, the interneuron populations exhibited different effects on epileptiform discharges. Recruiting VIP interneurons did not change the total duration of epileptiform discharges. In contrast, recruiting SST or PV interneurons produced robust suppression of epileptiform synchronization. PV interneurons exhibited the strongest effect per cell, eliciting at least a fivefold greater reduction in epileptiform activity than the other cell types. Consistent with this, we found that *in vivo* chemogenetic recruitment of PV interneurons suppressed convulsive behaviors by more than 80%. Our findings support the idea that selective chemogenetic enhancement of inhibitory synaptic pathways offers potential as an anti-seizure strategy.

## Introduction

Drugs that enhance GABA-mediated synaptic inhibition can be potent anticonvulsants (Czapiński et al., 2005). However, because of their system-wide actions, these drugs exhibit multiple deleterious side-effects (Snodgrass, 1992; Mula, 2011). While GABAergic signaling can become altered in cells within the epileptic focus (Cohen et al., 2002; Huberfeld et al., 2007), inhibitory mechanisms remain effective within the ‘penumbra’ surrounding the epileptic focus and are able to oppose seizure spread (Trevelyan et al., 2006, 2007; Schevon et al., 2012; Cammarota et al., 2013). Selectively enhancing these endogenous inhibitory mechanisms therefore offers the potential to disrupt the propagation of epileptic discharges.

The region of the brain that often contains the epileptic focus in temporal lobe epilepsy patients – the hippocampus – includes multiple subtypes of GABA-releasing interneurons, which are thought to vary in terms of their inhibitory capacity (Klausberger et al., 2003). For instance, because of their intrinsic properties and perisomatic targeting of multiple postsynaptic pyramidal neurons, parvalbumin-expressing (PV) interneurons have been considered particularly effective at inhibiting principal neurons (Cobb et al., 1995; Freund and Buzsáki, 1996; Miles et al., 1996) and at restricting the propagation of network activity (Trevelyan et al., 2006; Cammarota et al., 2013). Meanwhile, because of their post-synaptic targeting, somatostatin-expressing (SST) interneurons have been associated with the regulation of dendritic excitability (Miles et al., 1996; Paz and Huguenard, 2015), which can then affect the spiking output of principal neurons (Lovett-Barron et al., 2012). Other interneuron subtypes, such as vasoactive intestinal polypeptide-expressing (VIP) interneurons, can mediate disinhibitory effects as well as inhibitory effects, apparently because many of their postsynaptic targets are interneurons (Acsády et al., 1996).

Consistent with these ideas, studies using optogenetic strategies to increase interneuron activity have reported promising results in terms of reducing seizure activity (Krook-Magnuson et al., 2013; Ledri et al., 2014) and have indicated that interneuron subtypes can exert differential effects upon seizure generation and progression (Cammarota et al., 2013; Krook-Magnuson et al., 2013; Sessolo et al., 2015; Khoshkhoo et al., 2017). However, the temporally synchronous nature of optical activation can also generate counterintuitive effects, as the simultaneous recruitment of interneurons can enhance network synchronization and actually initiate epileptiform activity (Sessolo et al., 2015; Yekhlef et al., 2015; Chang et al., 2018).

A novel alternative strategy is afforded by chemogenetic tools such as Designer Receptors Exclusively Activated by Designer Drugs (DREADDs), which use pharmacological agents to enhance or inhibit the activity of defined cell populations (Armbruster et al., 2007; Alexander et al., 2009). DREADDs are mutated human muscarinic receptors that can be expressed in a cell-specific manner and are not activated by endogenous ligands, but are activated by drugs such as clozapine *N*-oxide (CNO) and related metabolites (Gomez et al., 2017). Activating excitatory DREADDs, such as the human type-3 muscarinic designer receptor coupled with the G_q_ protein (hM_3_D_q_ receptor), is thought to enhance neuronal excitability by downregulating ion channels that hyperpolarize the membrane (Alexander et al., 2009).

Excitatory hM_3_D_q_ DREADDs have been used to activate interneurons in several brain regions (Hamm and Yuste, 2016; Chen et al., 2017; Wang et al., 2017). However, it remains unclear to what extent different subtypes of GABAergic interneurons can be modulated via DREADDs, and whether chemogenetic control of different interneurons is a viable strategy to reduce epileptiform activity. Here, we demonstrate that three of the major interneuron subtypes in the hippocampus (PV-, SST-, and VIP-expressing interneurons) can be successfully recruited with excitatory DREADDs. The subtypes differ however, in their capacity to increase post-synaptic inhibition in principal neurons and ability to reduce epileptiform synchronization of the neuronal network. By confirming results across *in vitro* and *in vivo* models of both chronic and acute seizure activity, our study suggests that chemogenetically enhancing specific interneuron populations may offer an effective anticonvulsant strategy.

## Materials and Methods

### Preparation and Viral Transduction of Organotypic Hippocampal Brain Slices

All animal work relating to *in vitro* preparations was carried out in accordance with the Animals (Scientific Procedures) Act, 1986 (United Kingdom) and under project and personal licenses approved by the Home Office (United Kingdom). Mouse organotypic hippocampal brain slice cultures were prepared from 5- to 7-day-old heterozygous or homozygous, male or female PV-cre mice (B6; 129P2-Pvalb^tm1(cre)Arbr^/J, The Jackson Laboratory), SST-IRES-cre mice (Sst^tm2.1(cre)Zjh^/J, The Jackson Laboratory), or VIP-IRES-cre mice (Vip^tm1(cre)Zjh^/J, The Jackson Laboratory), as described by Stoppini et al. (1991). All reagents were purchased from Sigma-Aldrich, unless stated otherwise. The brains were extracted and transferred into cold (4°C) dissection media containing Earle’s Balanced Salt Solution + CaCl_2_ + MgSO_4_ (Thermo Fisher Scientific), supplemented with 25.5 mM HEPES, 36.5 mM D-glucose, and 5 mM NaOH. The hemispheres were separated, and the individual hippocampi were dissected and immediately sectioned into 400-μm-thick slices on a McIlwain tissue chopper (Mickle, United Kingdom). Slices were then rinsed in cooled dissection media, placed in 6-well plates onto sterile, porous Millicell-CM membranes, and maintained for 2–8 weeks in culture media containing 78.8% (vol/vol) Minimum Essential Media + GlutaMAX-I (Thermo Fisher Scientific), 20% (vol/vol) heat-inactivated horse serum (Thermo Fisher Scientific), 1% (vol/vol) B27 (Thermo Fisher Scientific), 30 mM HEPES, 26 mM D-glucose, 5.8 mM NaHCO_3_, 1 mM CaCl_2_, 2 mM MgSO_4_⋅7H_2_O, and incubated at 35.5–36°C in a 5% CO_2_ humidified incubator.

After 3–5 days in culture, organotypic hippocampal slices were transduced with adeno-associated virus (AAV, serotype 8) containing loxP-flanked, inverted DNA sequences under the control of the human Synapsin 1 promoter (University of North Carolina Gene Therapy Center Vector Core and Addgene, United States). Viral DNA contained the double-floxed sequence for hM_3_D_q_-mCherry (Addgene #44361), which was used to target the excitatory DREADDs to specific cre-expressing populations. In control experiments and to determine interneuron expression profiles, viral DNA contained the double-floxed sequence for EGFP (Addgene #50457). Transduction was achieved by injecting viral particles (mixed with 1% wt/vol fast-green for visualization) into 5–10 locations along the pyramidal cell layer of the hippocampal slices. Injection pipettes were pulled from glass capillaries (1.2 mm outer diameter, 0.69 mm inner diameter; Warner Instruments) using a horizontal puller (Sutter P-97), mounted on a manual manipulator (Narishige, Japan) and monitored under a microscope (Leica S6E) coupled with an external fiber optic light source (Photonic Leica CLS 100X). A Picospritzer II system (General Valve) delivered controlled pressure pulses (5–10 psi for 1 s) to facilitate gradual diffusion of the viral solution into the tissue. Typical titres were ∼10^12^ IU/ml and injection volumes were ∼250 nL per slice. Feeding media was supplemented with 1% (vol/vol) antibiotic and antimycotic solution (with 10,000 units penicillin, 10 mg streptomycin and 25 μg amphotericin B per mL) for up to two feeding sessions after injection and slices were allowed at least 2 weeks for expression before being used.

### Electrophysiological Recordings *in vitro*

The organotypic hippocampal slices were transferred to a recording chamber, where they were maintained at 28°C and continuously superfused with artificial cerebrospinal fluid (aCSF) containing (in mM): NaCl (120), KCl (3), MgCl_2_ (0.5–1.5), CaCl_2_ (2–3), NaH_2_PO_4_ (1.2), NaHCO_3_ (23), D-glucose (11) and ascorbic acid (0.2). Osmolarity was adjusted to 290 mOsm and pH was adjusted to 7.36 with NaOH. Oxygen and pH levels were stabilized by bubbling the aCSF with 95% O_2_ and 5% CO_2_. Neurons within the hippocampal formation were visualized with 10× and 60× water-immersion microscope objectives (Olympus BX51WI) and targeted for single or dual-patch whole-cell recordings. Patch pipettes of 4–9 MΩ tip resistance were pulled from filamental borosilicate glass capillaries with an outer diameter of 1.2 mm and an inner diameter of 0.69 mm (Warner Instruments), using a horizontal puller (Sutter P-97). For current clamp recordings, pipettes were filled with a potassium-gluconate internal solution (134 mM K-gluconate, 2 mM NaCl, 10 mM HEPES, 2 mM Na_2_ATP, 0.3 mM NaGTP, 2 mM MgATP), which had been set to a pH of 7.36 using KOH, and an osmolarity of 290 mOsm. For recording post-synaptic inhibitory currents in voltage clamp, pipettes were filled with a caesium-gluconate internal solution (120 mM Cs-gluconate, 4 mM NaCl, 40 mM HEPES, 2 mM MgATP, 0.3 mM NaGTP, and 0.2 mM QX-314). Before use, internal solutions were filtered with a 0.22 μm syringe filter (Merck Millipore). Pipettes were mounted to a headstage (CV-7b, Molecular Devices, United States) and controlled via a Multiclamp 700B amplifier (Axon CNS, Molecular Devices). Following entry into whole cell configuration, access resistance (R_a_) was monitored every 2 min and experiments were only included if R_a_ remained stable and below 25 MΩ. Recordings were low-pass filtered online at 2 kHz (8-pole Bessel), acquired using Clampex software (pClamp 10, Molecular Devices), and exported into MATLAB (R2017a, Mathworks) for offline analysis using custom-made scripts.

To examine the direct effects of activating excitatory DREADDs upon interneuron excitability, current clamp recordings were conducted in aCSF containing kynurenic acid (3 mM) and hM_3_D_q_ receptors were activated by bath application of CNO (10–20 μM, Tocris, Bio-Techne). The spontaneous action potential firing rate of each interneuron was compared for a 5-min period before and after CNO application, having allowed 3 min for the CNO to reach the chamber. To measure the post-synaptic GABAergic currents induced by activating hM_3_D_q_ receptors in a specific interneuron population, voltage-clamp recordings were conducted by clamping CA1 and CA3 pyramidal neurons at the reversal potential for glutamatergic current (E_GLUT_) in the presence of kynurenic acid. Once recordings had stabilized, the amplitude of post-synaptic inhibitory conductances were compared across 2-min periods recorded under baseline conditions, after bath application of CNO and then after co-administration of CNO and tetrodotoxin (TTX, 1–2 μM).

### Quantification of Epileptiform Discharges *in vitro*

A semi-automated detection algorithm was used to identify the start and end of individual epileptiform discharges (EDs) *in vitro*. Current-clamp traces were down-sampled to 1 kHz and then band-pass filtered (typically 0.05–0.2 Hz) using a Bessel filter (2nd order). The signal was corrected for the rise time of the filter and subsequently rectified, thresholded and binarized, merging events that were close in time (typically under 1 min apart), and ignoring events shorter than 5 s. Experiments to test the effects of a drug (e.g., CNO) comprised a 15-min baseline, followed by a 3-min period to allow the drug to reach the recording chamber, and a further 15-min period in which the slice was continuously superfused with drug-containing aCSF. The 15-min time periods (‘baseline’ and ‘drug’) were assessed using the same ED detection settings. Total ED activity was defined as the sum of time that the slice displayed ED activity during a 15-min period. ED frequency was calculated from the number of EDs that initiated during a 15-min period and ED length was the mean duration of individual EDs that were completely contained within a 15-min period.

### Viral Transduction of Hippocampal Interneurons *in vivo*

All animal procedures relating to *in vivo* experiments were carried out with the approval of the local ethics committee for animal research in Bucharest and in accordance with European Union Directive 2010/63/EU on the protection of animals used for scientific purposes. For viral injections, adult animals of either sex were anesthetized with isoflurane (maintained at 1.5–2%, 0.4 L/min) and mounted on a stereotaxic instrument (Kopf, RWD Life Science). The level of anesthesia was continuously monitored, eye drops (Corneregel, Bausch and Lomb) were applied to avoid corneal desiccation and a heat pad system (DC Temperature Controller, FHC) was used to maintain the body temperature in the physiological range. Wiretrol II glass capillaries (Drummond Scientific) were pulled using a vertical puller (Narishige PC-10, Japan) and connected to the Hamilton syringe via compression fittings (RN 1 mm, Hamilton, United States). Small craniotomies were generated with a precision drill (FBS 240/E, Proxxon Micromot) and hippocampal bilateral injections were performed both dorsally [-2.18 anteroposterior (AP), 2.3 mediolateral (ML), 2.4–1.85 dorsoventral (DV), relative to Bregma] and ventrally (-2.7 AP, 2.9 ML, 3.1–2.25 DV, relative to Bregma) at a rate of 1.66 nL/s, slowly retreating the injection pipette (0.91 μm/s) to maximize delivery throughout the hippocampus. The injection was controlled via a micromanipulator (NeuroCraft MCM, FHC) attached to a syringe (705RN, 50 μL, Hamilton, United States). Each of four injection tracks was infused with 1,350 nL of virus (Addgene, United States), of which 170 nL were delivered at each end of the track. After infusing the target volume of viral solution, a time window of 5 min allowed the virus solution to spread through the hippocampal tissue before the injection pipette was completely retracted. After allowing 2–6 months for expression, and at least 3 days before commencing seizure experiments, mice were anesthetized and implanted with an infusion cannula (C315GS-5 guide cannula, Plastics One, United States) directly over the viral injection site in the right dorsal hippocampus. The cannula was secured to the skull via bone cement (Refobacin R40, Biomet UK, United Kingdom).

### Quantification of Seizure Behavior *in vivo*

For each seizure experiment, mice were briefly anesthetized with isoflurane to allow insertion of the infusion cannula into the guide cannula, such that the tip of the infusion cannula was located within the hippocampus at -2.18 AP, 2.3 ML, 2.2 DV, relative to Bregma. At this point, an intraperitoneal (i.p.) bolus of solution containing CNO [4 mg/kg with 4% dimethyl sulfoxide (DMSO) in saline] or vehicle (4% DMSO in saline) was administered and the mouse was allowed to fully recover from anesthesia for 15 min before starting experiments. To monitor behavior, the mouse was placed in a square arena (400 mm by 400 mm) in which it was able to move freely virtue of a connector assembly (C313C, Plastics One, United States) and swivel system (375/22PS blue, 22ga, Instech, United States), which connected the infusion tubing to a 1 μL syringe (7101, Hamilton, United States) controlled by an infusion pump (IVAC P6000, Cardinal Health). Following a 20-min baseline period, 4-aminopyridine (4-AP) was infused directly into the hippocampus according to a spaced delivery protocol (three 4-AP infusions, each separated by 12 min and consisting of a 200 nL injection of 2 mM 4-AP over 2 min). Infusions were terminated immediately if the mouse reached the stage of generalized motor convulsions. Throughout each experiment video recordings were performed using two high speed, high definition cameras located at a right angle from each other (Hero 3+ Silver, GoPro, United States) at 60 frames per second, 1920 × 1080 pixels per frame. Polarized filters were used to reduce glare from the arena walls. A third camera captured the animals’ movements directly from above, allowing to track the location and locomotor activity of the mouse at any given time point. Seizure behavior was blindly scored using the Racine scale. Individual behaviors were considered as binary point events across time at a sampling frequency of 1 Hz. Racine 1 and 2 behaviors were classified as ‘non-convulsive’ events, and Racine 3, 4, and 5 behaviors were considered ‘convulsive’ events (Bergstrom et al., 2013; Tse et al., 2014; Sharma et al., 2018). For the cumulative convulsive Racine index, convulsive events were weighted according to the Racine classification of the behavior: (1) limb clonic activity (Racine 3); (2) retreating/rearing with orofacial clonic activity (Racine 4); (3) rearing and falling and/or jumping (Racine 5 – full motor convulsions).

### Immunohistochemistry and Quantification of Interneuron Distribution

For *in vitro* studies, organotypic hippocampal slices expressing hM_3_D_q_-mCherry or EGFP in specific interneuron populations were fixed overnight at 4°C in 4% paraformaldehyde with 4% sucrose, in 0.01 M phosphate buffer solution (PBS), pH 7.4. The slices used for immunofluorescence were washed and embedded in 3% agar, and re-sectioned at 50 μm on a vibrating microtome (Microm HM 650V, Thermo Fisher Scientific). For *in vivo* studies, mice expressing hM_3_D_q_-mCherry were transcardially perfused and the brains sectioned at 50 μm. PV expression was visualized by incubating sections in 1:500 guinea pig primary antibody (cat. no. 195 004, Synaptic Systems) in PBS with 0.3% Triton-X (PBST) with 1% normal goat serum (NGS, Thermo Fisher Scientific) overnight at 4°C, followed by 1:500 Alexa 488 goat anti-guinea pig secondary antibody (Thermo Fisher Scientific) in PBST with 1% NGS overnight at 4°C. For SST immunolabeling, the tissue was processed with a basic antigen retrieval kit at 95°C for 10 min (R&D Systems). All sections were pre-incubated in 10% NGS in PBST for at least 2 h at room temperature. SST expression was visualized by incubating sections in 1:250 rat primary antibody (MAB 354, Millipore) in PBST with 1% NGS for 11 days at 4°C, followed by 1:500 Alexa 488 goat anti-rat secondary antibody (Thermo Fisher Scientific) in PBST with 1% NGS for 2 days at 4°C. VIP expression was visualized by incubating sections in 1:5000 rabbit primary antibody (donation from Professor Peter P. Somogyi) in PBST with 1% NGS for 2 days at 4°C, followed by 1:500 Alexa 488 goat anti-rabbit secondary antibody (Thermo Fisher Scientific) in PBST with 1% NGS overnight at 4°C. Finally, all sections were mounted in Vectashield (Vector Laboratories) and images were captured with an LSM 880 confocal microscope equipped with 488 nm and 561 nm lasers, a 20× water-immersion objective (W Plan-Apochromat, NA 1.0) and controlled via the ZEN black software (Zeiss).

To determine the number and distribution of soma and processes associated with each interneuron subtype, we performed quantitative image analysis on organotypic hippocampal slices from PV-cre, SST-cre, and VIP-cre mice that had been injected with floxed AAVs. Slices were subjected to confocal microscopy and, in the resulting images, the CA areas were linearized along the pyramidal layer and the soma of fluorescent neurons were automatically detected via a custom-made, two-pass algorithm extracting maximally stable extremal region features using MATLAB (Matas et al., 2004; Nistér and Stewénius, 2008). The number of virally transduced interneurons was derived directly from the number of fluorescent soma per optical section. Meanwhile, to describe the distribution of processes associated with each interneuron subtype, soma were digitally removed from the linearized images to generate a transverse expression profile of fluorescent processes relative to the pyramidal cell layer. To compare across multiple interneuron populations, these expression profiles were normalized by the area under each curve.

### Data Analysis

Digital signal processing and presentation were performed using custom-made programs in the MATLAB environment (R2017b, Mathworks). Figures were built using vector-based graphic design in CorelDraw (X6, Corel Corporation) and the statistical software GraphPad Prism (v6.01, GraphPad Software). Video data processing for tracking locomotor activity of animals was performed using the open-source software Bonsai v2.3 (Lopes et al., 2015). Data are presented as mean ± standard error of mean, and the statistical tests are reported at the relevant points in the text (GraphPad Prism; MATLAB). Non-parametric tests were used when a normal distribution of data could not be ascertained. Appropriate *post hoc* tests were used when ANOVA tests confirmed a statistically significant effect.

## Results

### Recruiting Distinct Hippocampal GABA-Releasing Interneuron Populations With Excitatory DREADDs

**FIGURE 1:**
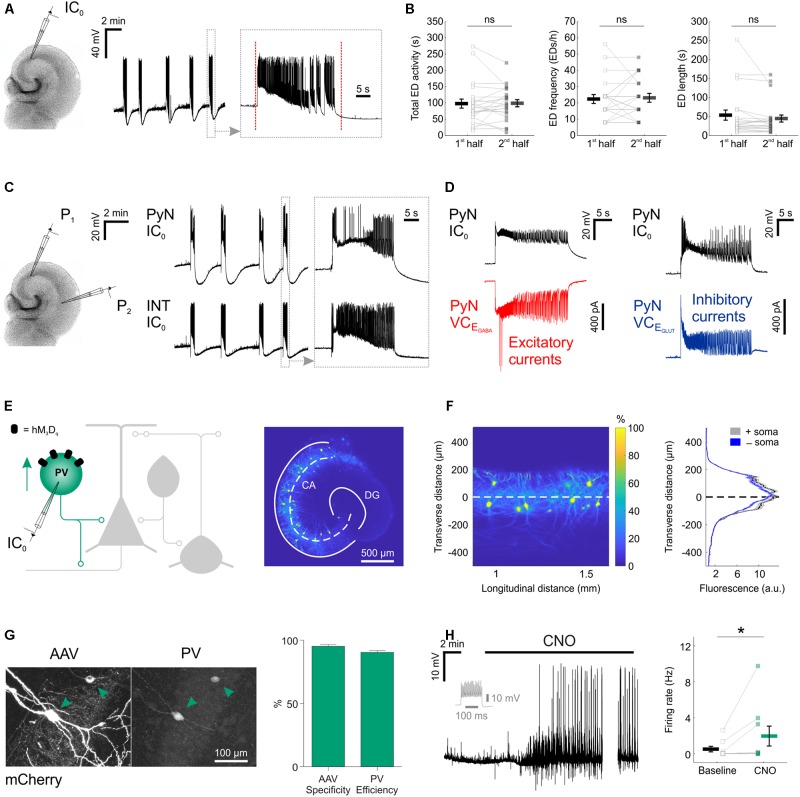
Chemogenetic recruitment of hippocampal interneurons in an *in vitro* model of epileptiform activity. **(A)** Current-clamp recording of a CA3 pyramidal neuron from a mouse organotypic hippocampal slice reveals repeated EDs. The vertical red dotted lines in the expanded trace mark the onset and the termination of the underlying ED, as determined by an automated detection algorithm. **(B)** A 15-min time window was used to assess the stability of the epileptiform activity. Total ED activity, defined as the cumulative duration of ED activity [left, 96.8 ± 13.9 s during 1st half, 97.9 ± 10.9 s during 2nd half; *N* = 22 slices, *W*_(21)_ = 110.5, *p* = 0.8715, two-tailed Wilcoxon signed-rank test], ED frequency [middle, 22.2 ± 2.7 EDs/h during 1st half, 22.9 ± 2.7 EDs/h during 2nd half; *N* = 22 slices, *W*_(12)_ = 34, *p* = 0.7153, two-tailed Wilcoxon signed-rank test], and individual ED length [right, 52.8 ± 13.4 s during 1st half, 43.9 ± 9.2 s during 2nd half; *N* = 22 slices, *W*_(21)_ = 71, *p* = 0.1281, two-tailed Wilcoxon signed-rank test] were stable across the 15-min time window. **(C)** A dual current-clamp (IC_0_) electrophysiological recording of a CA3 pyramidal neuron (PyN) and a GABAergic interneuron (INT) reveal spontaneous EDs in a mouse organotypic hippocampal brain slice (left). Expanded traces (right) show that both excitatory and inhibitory neurons are recruited during the EDs. **(D)** Simultaneous current clamp and voltage clamp (VC) recordings from pairs of pyramidal neurons demonstrate that strong barrages of excitatory (bottom-left, red) and inhibitory (bottom-right, blue) post-synaptic currents occur throughout the EDs, as monitored by current clamp recordings from a neighboring pyramidal neuron (top). E_GABA_, reversal potential for GABAergic current; E_GLUT_, reversal potential for glutamatergic current. **(E)** Cartoon of hippocampal circuitry (left) showing the targeting of the hM_3_D_q_ receptor to PV interneurons. Confocal image (right) of a hippocampal slice from a PV-cre mouse illustrates the fluorescence distribution profile of virally transduced PV interneurons. The superimposed white dashed line marks the center of the pyramidal cell layer. Continuous white lines indicate the dentate gyrus (DG) and outline of the CA areas (CA). **(F)** The confocal image was linearized (left) to facilitate the quantification of the transverse expression profile for PV interneurons, relative to the pyramidal cell layer (dashed line at zero). This confirmed that PV interneurons (+soma) and their processes (-soma) were restricted to the pyramidal cell layer (right). **(G)** The immunohistochemical characterization of PV interneurons transduced with AAV_8_-hSyn-DIO-hM_3_D_q_-mCherry demonstrates high targeting specificity and efficiency (*N* = 14 sections from six slices). **(H)** An example current-clamp recording of a PV interneuron expressing hM_3_D_q_ receptors shows that CNO promotes action potential firing (left). The firing pattern of the neuron in response to a 500 pA square wave current pulse is shown in gray. The break in the recording reflects a period during which the intrinsic properties of the neuron were monitored. Recordings were conducted in kynurenic acid to isolate the direct effects of hM_3_D_q_ receptor activation, and to prevent the occurrence of EDs. Population data shows a significant increase in firing rate following CNO [right; *N* = 9 slices, *W*_(7)_ = 0, *p* = 0.0156, two-tailed Wilcoxon signed-rank test]. ^∗^*p* < 0.05.

**FIGURE 2:**
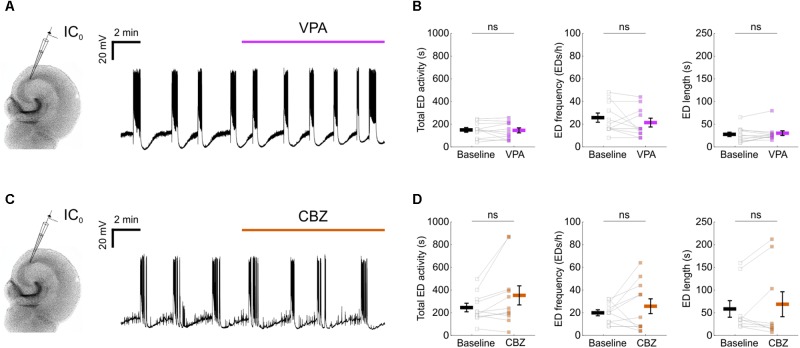
Epileptiform activity in mouse organotypic hippocampal brain slices exhibits resistance to first-line anti-epileptic drugs. **(A)** A representative recording showing that 1.5 mM valproic acid (VPA) did not disrupt the spontaneous EDs. **(B)** No impact could be detected of VPA on the total ED activity [left, 146.6 ± 20.4 s during baseline and 142.6 ± 22 s during VPA; *N* = 11, *t*_(10)_ = 0.31, *p* = 0.7643, two-tailed paired *t*-test], frequency [middle, 25.5 ± 4 EDs/h during baseline and 21.1 ± 3.9 EDs/h during VPA; *N* = 11, *t*_(10)_ = 1.17, *p* = 0.2674, two-tailed paired *t*-test] or individual ED length [right, 27 ± 5 s during baseline and 29.5 ± 5.2 s during VPA; *N* = 11, *W*_(11)_ = 22, *p* = 0.3652, two-tailed Wilcoxon signed-rank test]. **(C)** A representative recording showing that 50 μM carbamazepine (CBZ) did not disrupt the spontaneous EDs. **(D)** No impact could be detected of CBZ on the total ED activity [left, 242.7 ± 37.8 s during baseline and 350.3 ± 84.2 s during CBZ; *N* = 11, *t*_(10)_ = 2.136, *p* = 0.0584, two-tailed paired *t*-test], frequency [middle, 19.6 ± 2.7 EDs/h during baseline and 25.5 ± 6.5 EDs/h during CBZ; *N* = 11, *t*_(10)_ = 1.03, *p* = 0.3266, two-tailed paired *t*-test] or individual ED length [right, 57.8 ± 18.3 s during baseline and 68.1 ± 27.4 s during CBZ; *N* = 9, *W*_(9)_ = 21, *p* = 0.9102, two-tailed Wilcoxon signed-rank test]. VPA and CBZ drug concentrations were equivalent to effective doses *in vivo* (Albus et al., 2008).

To examine the potential of enhancing the synaptic output of hippocampal interneurons chemogenetically, we used mouse organotypic hippocampal brain slices. This system enabled us to perform targeted patch clamp recordings to determine the pre-synaptic and post-synaptic efficacy of DREADDs, as well as the opportunity to examine the impact of interneuron recruitment upon spontaneously generated epileptiform activity. Organotypic hippocampal brain slices can be used as a model of temporal lobe epilepsy because when kept in culture beyond 2 weeks, they develop spontaneous EDs without any pharmacological treatment, analogous to epileptogenesis in post-traumatic epilepsy (Dyhrfjeld-Johnsen et al., 2010; Lillis et al., 2015). As in previous work (Trevelyan et al., 2006; Sessolo et al., 2015), our recordings from excitatory pyramidal neurons within the Cornu Ammonis (CA) areas revealed reproducible spontaneous EDs that exhibited a sustained duration (mean duration 48.3 ± 8 s) and stable frequency (**Figures 1A,B**). Consistent with epileptiform activity in many systems (Trevelyan et al., 2006; Sessolo et al., 2015), these spontaneous EDs recruited both excitatory and inhibitory neurons within the network. Paired recordings revealed that during each ED, pyramidal neurons received intense barrages of both glutamatergic and GABAergic post-synaptic currents (**Figures 1C,D**). To further characterize EDs, we tested the effect of first-line anti-epileptic drugs, valproate and carbamazepine. At therapeutically relevant doses, both drugs were ineffective at suppressing EDs (**Figure 2**), consistent with the idea that organotypic slices represent a model of drug-resistant temporal lobe epilepsy (Albus et al., 2008; Avaliani et al., 2016). By combining promoter-specific cre-recombinase mice with floxed chemogenetic constructs, we were then able to investigate the cellular and network effects of delivering DREADDs to specific interneuron populations.

Parvalbumin interneurons, a principal GABAergic population, reside primarily within the pyramidal layer of CA areas (Pawelzik et al., 2002), and their axons target the somatic compartment of pyramidal neurons (Pawelzik et al., 2002; Bartos and Elgueta, 2012). Consistent with this, injection of AAV containing floxed constructs into organotypic hippocampal slices from PV-cre mice resulted in somatic and process expression that was restricted to the pyramidal cell layer (**Figures 1E,F**). Immunohistochemical experiments confirmed that the PV interneurons could be efficiently and specifically targeted with the excitatory DREADD receptor, hM_3_D_q_. Two to four weeks after viral transduction with AAV_8_-hSyn-DIO-hM_3_D_q_-mCherry, the majority of expressing neurons were immunopositive for PV (95.2 ± 1.5% ‘specificity’) and the majority of all PV immunopositive neurons were expressing hM_3_D_q_-mCherry (90.5 ± 1.4% ‘efficiency’; **Figure 1G**). To assess whether activating hM_3_D_q_ receptors could increase the output of the PV interneuron population, current clamp recordings were targeted to hM_3_D_q_-mCherry positive neurons. The addition of the hM_3_D_q_ ligand, CNO, led to a significant increase in the firing rate of PV interneurons, from 0.5 ± 0.3 Hz during baseline, to 1.9 ± 1.1 Hz in the presence of CNO (**Figure 1H**).

**FIGURE 3:**
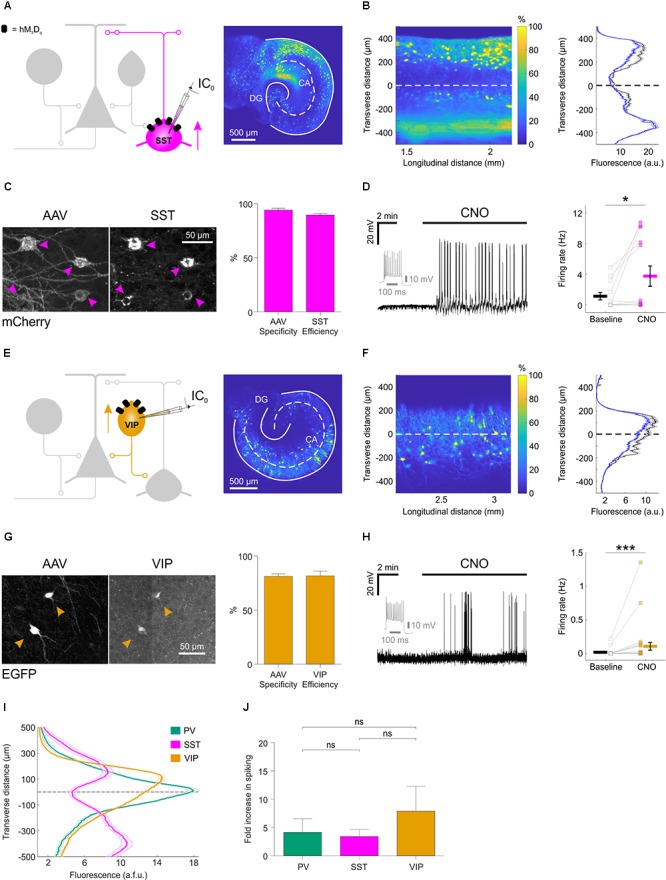
Distinct subtypes of hippocampal GABAergic interneurons can be recruited via excitatory DREADDs. **(A)** Cartoon (left) showing the targeting of hM_3_D_q_ receptors to SST hippocampal interneurons. A confocal image of a SST-cre mouse slice (right) illustrates the fluorescence distribution profile of virally transduced SST interneurons. Continuous white lines outline the DG and CA areas. **(B)** The confocal image was linearized (left) to facilitate quantification of the transverse expression profile for SST interneurons, relative to the pyramidal cell layer (dashed white line at zero). This confirmed that SST interneurons (+soma) and their processes (-soma) were associated with stratum oriens and lacunosum-moleculare (right). **(C)** Immunohistochemical characterization of SST interneurons transduced with AAV_8_-hSyn-DIO-hM_3_D_q_-mCherry demonstrates high targeting specificity and efficiency (*N* = 16 sections from six slices). **(D)** Current-clamp recording from a SST interneuron expressing hM_3_D_q_ in the presence of kynurenic acid, showing that CNO promotes action potential firing (left). The firing pattern of the neuron in response to a 500 pA square wave current pulse is shown in gray. Population data shows a significant increase in firing rate in the presence of CNO [right, *N* = 13 slices, *W*_(9)_ = 4, *p* = 0.0273, two-tailed Wilcoxon signed-rank test]. **(E)** Cartoon (left) shows the targeting of hM_3_D_q_ receptors to VIP interneurons and confocal image (right) illustrates the fluorescence distribution profile of virally transduced interneurons in a VIP-cre slice. **(F)** Linearizing the confocal image (left) confirmed that the expression profile for VIP interneurons (+soma) and their processes (-soma) was associated with stratum radiatum/lacunosum-moleculare, pyramidale and oriens (right). **(G)** Immunohistochemical characterization of VIP interneurons transduced with AAV_8_-hSyn-DIO-hM_3_D_q_-mCherry demonstrates high targeting specificity and efficiency (*N* = 4 sections from two slices). **(H)** Example current-clamp recording from a VIP interneuron expressing hM_3_D_q_ in kynurenic acid, showing that CNO promotes action potential firing (left). The firing pattern of the neuron in response to a 500 pA square wave current pulse is shown in gray. Population data (right) shows a significant increase in firing rate in the presence of CNO [*N* = 27 slices, *W*_(13)_ = 1, *p* = 0.0005, two-tailed Wilcoxon signed-rank test]. **(I)** Comparison of the fluorescence distribution profiles following viral-transduction of the three interneuron populations [PV, *N* = 38 slices; SST, *N* = 37 slices; VIP, *N* = 57 slices]. Each distribution was normalized by the area under the profile curve and shown to be significantly different between the cell types [interaction between cell type and location: *F*_(8,516)_ = 58.81; *p* < 0.0001, repeated measures two-way ANOVA]. **(J)** There was no difference in the CNO-induced fold-increase in spiking rate (normalized to baseline) between the three interneuron populations [χ^2^_(2)_ = 0.9136, *p* = 0.6333, Kruskal–Wallis test]. ^∗^*p* < 0.05, ^∗∗∗^*p* < 0.001.

We performed similar experiments in slices generated from SST-cre mice and VIP-cre mice (**Figure 3**). Consistent with previous evidence that SST interneurons target the dendritic compartments of hippocampal principal neurons (Katona et al., 1999; Lovett-Barron et al., 2012), we found that the soma and processes of SST interneurons were located within stratum oriens and lacunosum-moleculare, and tended to avoid the pyramidal cell layers (**Figures 3A,B**). Immunohistochemistry confirmed that the SST interneurons were efficiently (89.6 ± 1.5%) and specifically (94.1 ± 1.6%; **Figure 3C**) targeted with hM_3_D_q_ receptors. Consistent with the results in PV interneurons, CNO significantly increased the firing rate of SST interneurons, from 1.1 ± 0.5 Hz during baseline, to 3.7 ± 1.3 Hz (**Figure 3D**).

Consistent with previous evidence that distinct VIP interneuron subtypes target oriens lacunosum-moleculare interneurons and the perisomatic regions or proximal dendrites of pyramidal neurons (Léránth et al., 1984; Acsády et al., 1996; Chamberland et al., 2010; Tyan et al., 2014) (**Figure 3E**), we found that the soma and processes of virally transduced VIP interneurons were located within stratum radiatum/lacunosum-moleculare, pyramidale and oriens in slices from the VIP-cre mice (Köhler, 1982; Taniguchi et al., 2011) (**Figure 3F**). The majority of expressing neurons were immunopositive for VIP (81.2 ± 2.4% specificity) and the majority of VIP immunopositive neurons expressed the floxed construct (81.6 ± 4.4% efficiency; **Figure 3G**). Finally, CNO increased the firing rate from 0.01 ± 0.01 Hz during baseline, to 0.1 ± 0.1 Hz (**Figure 3H**).

A summary plot for the three interneuron populations confirmed that the expression profiles of PV, SST, and VIP interneurons were significantly different from one another (**Figure 3I**) and were consistent with data from acute preparations of mouse hippocampus (Taniguchi et al., 2011; Lovett-Barron et al., 2012). These results are in accordance with previous observations that organotypic slices retain fundamental features of the circuit, including the subcellular targeting of perisomatic and dendritic domains of pyramidal neurons by distinct interneuron populations (Streit et al., 1989; De Simoni et al., 2003; Cristo et al., 2004). Meanwhile, CNO-mediated activation of hM_3_D_q_ receptors resulted in comparable fold-increases in spiking activity across the three interneuron populations: PV interneurons showed a 4.1 ± 2.4 fold increase in their firing rate, SST interneurons showed a 3.4 ± 1.2 fold increase and VIP interneurons showed a 7.9 ± 4.4 fold increase (**Figure 3J**).

### Chemogenetic Enhancement of GABAergic Interneuron Populations Can Attenuate Epileptiform Activity *in vitro*

**FIGURE 4:**
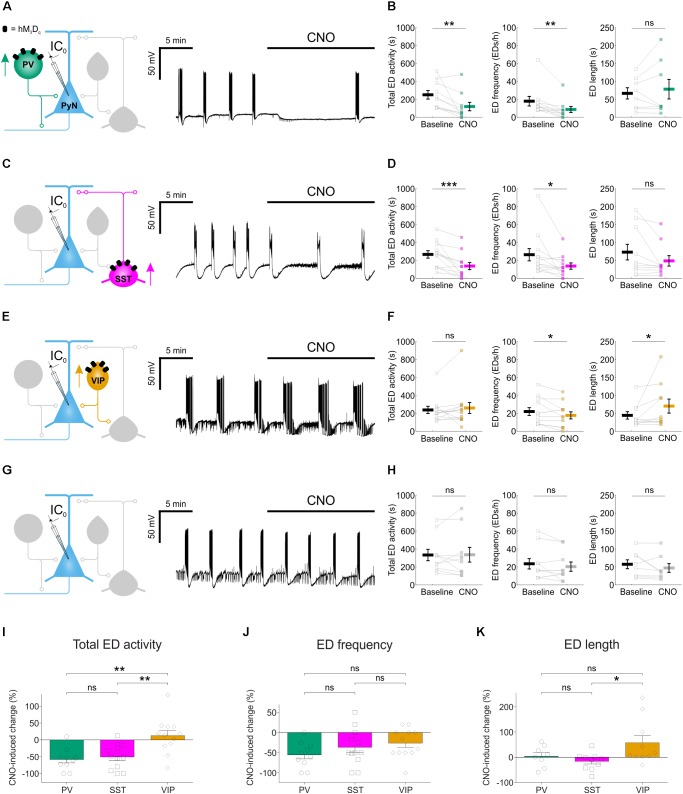
Chemogenetic enhancement of specific GABAergic interneuron populations attenuates hippocampal EDs. **(A)** PV-cre mice and floxed viral constructs were used to target hM_3_D_q_ receptors to PV interneurons in organotypic hippocampal brain slices. The effects of CNO upon spontaneous EDs were monitored by current-clamp recordings from CA1 or CA3 pyramidal neurons. **(B)** Population data from experiments targeting PV interneurons (*N* = 10 slices) showed a reduction in total ED activity [left, *W*_(10)_ = 1, *p* = 0.0039, two-tailed Wilcoxon signed-rank test], a decrease in ED frequency [middle, *W*_(9)_ = 0, *p* = 0.0039, two-tailed Wilcoxon signed-rank test], and no change in ED length [right, *W*_(8)_ = 12, *p* = 0.4609, two-tailed Wilcoxon signed-rank test] following addition of CNO. **(C)** SST-cre mice were used to target hM_3_D_q_ receptors to SST interneurons. **(D)** Population data from experiments targeting SST interneurons (*N* = 12 slices) showed a reduction in total ED activity [left, *W*_(12)_ = 1, *p* = 0.001, two-tailed Wilcoxon signed-rank test], a decrease in ED frequency [middle, *W*_(12)_ = 6, *p* = 0.0166, two-tailed Wilcoxon signed-rank test], and no change in ED length [right, *W*_(10)_ = 10, *p* = 0.0840, two-tailed Wilcoxon signed-rank test] following addition of CNO. **(E)** VIP-cre mice were used to target hM_3_D_q_ receptors to VIP interneurons. **(F)** Population data from experiments targeting VIP interneurons (*N* = 12 slices) showed no change in total ED activity [left, *W*_(12)_ = 25, *p* = 0.3013, two-tailed Wilcoxon signed-rank test], a reduction in ED frequency [middle, *t*_(11)_ = 2.24, *p* = 0.0468, two-tailed paired *t*-test], and an increase in ED length [right, *W*_(10)_ = 6, *p* = 0.0273, two-tailed Wilcoxon signed-rank test] following addition of CNO. **(G)** Control experiments were conducted on slices that had not received floxed DREADD constructs. **(H)** Population data (*N* = 10 slices) demonstrated no change in total ED activity [left, *t*_(9)_ = –0.08, *p* = 0.9337, two-tailed paired *t*-test], ED frequency [middle, *t*_(9)_ = 1.31, *p* = 0.2229, two-tailed paired *t*-test], or ED length [right, *W*_(8)_ = 6, *p* = 0.1094, two-tailed Wilcoxon signed-rank test] following the addition of CNO to control slices. **(I)** Total ED activity was significantly reduced by chemogenetically enhancing either PV or SST interneurons, compared to enhancing VIP interneurons [*F*_(2,31)_ = 9.747, *p* = 0.0005, one-way ANOVA, followed by Tukey’s *post hoc* multiple comparison tests; VIP vs. PV, *p* = 0.0013; VIP vs. SST, *p* = 0.0025]. **(J)** The frequency of EDs was reduced when PV interneurons, SST interneurons or VIP interneurons were targeted. No significant difference in the reduction of ED frequency was detected across the three interneurons [*F*_(2,31)_ = 1.583, *p* = 0.2215, one-way ANOVA]. **(K)** Individual EDs became significantly longer when VIP interneurons were recruited, compared to SST interneurons [*F*_(2,25)_ = 3.772, *p* = 0.037, one-way ANOVA, followed by Tukey’s *post hoc* multiple comparison tests; VIP vs. SST, *p* = 0.0337]. ^∗^*p* < 0.05, ^∗∗^*p* < 0.01.

We next tested the effects of enhancing the activity of each interneuron population on EDs. Spontaneous EDs were recorded from pyramidal neurons before and after CNO-mediated activation of the relevant interneuron population. We quantified total ED activity, ED frequency and individual ED duration. In PV-targeted slices (**Figure 4A**), CNO-mediated activation of hM_3_D_q_ receptors significantly reduced the total ED activity (from 247.5 ± 47.8 s during baseline to 116.5 ± 47.4 s during CNO), which resulted from a significant decrease in ED frequency (from 17.6 ± 5.3 EDs/h during baseline to 8.4 ± 3.3 EDs/h during CNO), without affecting the duration of individual EDs (65.8 ± 15.7 s during baseline and 77.5 ± 27.4 s during CNO) (**Figure 4B**). In SST-targeted slices (**Figure 4C**), CNO also significantly reduced total ED activity (from 263.2 ± 41.4 s during baseline to 133.3 ± 40.2 s during CNO), which involved a decrease in ED frequency (from 26 ± 6.8 EDs/h during baseline to 13.3 ± 3.5 EDs/h during CNO), without significantly affecting individual ED length (72 ± 21.7 s during baseline and 47.6 ± 14.5 s during CNO) (**Figure 4D**). In VIP-targeted slices (**Figure 4E**), CNO did not decrease the total ED activity (234.3 ± 40.6 s during baseline and 256.3 ± 62.7 s during CNO). While enhancing VIP interneuron output led to a decrease in ED frequency (from 21.7 ± 4.3 EDs/h during baseline to 17.3 ± 4 EDs/h during CNO), there was a simultaneous increase in the duration of individual EDs (from 43.5 ± 10.3 s during baseline to 69.4 ± 19.6 s during CNO) (**Figure 4F**). Finally, to assess potential off-target effects of CNO, the drug was bath-applied during the recording of spontaneous EDs in control slices virally transduced with the same AAV type expressing eGFP in a cre-dependent manner, and not hM_3_D_q_ (**Figure 4G**). In these experiments, no changes were detected for total ED activity, ED frequency, or individual ED length (**Figure 4H**).

Comparisons across the different interneuron populations confirmed subtype-specific effects. Total ED activity was reduced by more than half following activation of either PV interneurons (down 58.2 ± 10.3%) or SST interneurons (down 50.8 ± 10.5%), and both of these reductions were significantly greater than the change in total ED activity induced by recruiting VIP interneurons (up 12.7 ± 15.4%) (**Figure 4I**). Each of the interneuron populations was able to decrease ED frequency: PV interneurons by 55.4 ± 10.1%, SST interneurons by 36.7 ± 13% and VIP interneurons by 26.3 ± 10.4% (**Figure 4J**). Meanwhile, only the VIP population increased individual ED length by 57.5 ± 28.4% (**Figure 4K**). In summary, chemogenetically enhancing the output of interneuron populations can generate effective suppression of epileptiform synchronization, but the effectiveness of this approach varied by interneuron subtype.

### Chemogenetically Enhanced Interneuron Subtypes Differ in Their Post-synaptic Inhibition of Pyramidal Neurons

**FIGURE 5:**
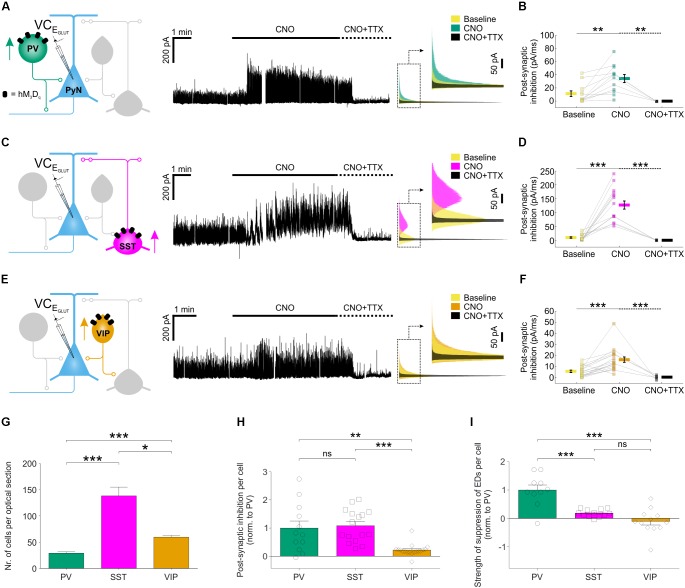
Chemogenetic recruitment of interneuron populations generates different amounts of post-synaptic inhibition in pyramidal neurons. **(A)** hM_3_D_q_ receptors were targeted to PV interneurons and voltage-clamp recordings (at E_GLUT_) were performed from pyramidal neurons (left). CNO application elicited a pronounced increase in inhibitory post-synaptic currents converging upon the pyramidal neuron (middle). This increase was associated with spiking activity as it was abolished by bath application of TTX. Overlapping histograms (right) illustrate the probability distribution functions for the inhibitory post-synaptic currents during baseline, CNO, and co-administration of CNO plus TTX. **(B)** PV recruitment resulted in a significant change in total post-synaptic inhibitory charge converging onto pyramidal neurons [*F*_(2,26)_ = 9.541, *p* = 0.0008, one-way ANOVA, followed by *post hoc* Sidak’s multiple comparisons tests; baseline vs. CNO, *N* = 12, *p* = 0.0047; CNO vs. CNO plus TTX, *N* = 5, *p* = 0.0013]. **(C)** hM_3_D_q_ receptors were targeted to SST interneurons and all conventions are the same as in **(A)**. **(D)** SST recruitment resulted in a significant change in total post-synaptic inhibitory charge converging onto pyramidal neurons [χ^2^_(2)_ = 29.77, *p* < 0.0001, Kruskal–Wallis test, followed by *post hoc* Dunn’s multiple comparisons tests; baseline vs. CNO, *N* = 16, *p* < 0.0001; CNO vs. co-administration of CNO plus TTX, *N* = 5, *p* < 0.0001]. **(E)** hM_3_D_q_ receptors were targeted to VIP interneurons and all conventions are the same as in **(A)**. **(F)** VIP recruitment resulted in a significant change in total post-synaptic inhibitory charge converging onto pyramidal neurons [*F*_(2,39)_ = 14.09, *p* < 0.0001 by one-way ANOVA, followed by *post hoc* Sidak’s multiple comparisons tests; baseline vs. CNO, *N* = 18, *p* = 0.0003; CNO vs. co-administration of CNO plus TTX, *N* = 6, *p* = 0.0001]. **(G)** The average number of hM_3_D_q_ expressing interneurons (per 24 μm optical section) differs significantly between the three interneuron populations, with an average of 29.1 ± 2.7 PV cells, 138.1 ± 16.8 SST interneurons, and 59.7 ± 3.2 VIP cells [χ^2^_(2)_ = 54.44, *p* < 0.0001, Kruskal–Wallis test, followed by Dunn’s multiple *post hoc* comparisons; PV vs. SST, *p* < 0.0001; PV vs. VIP, *p* < 0.0001; SST vs. VIP, *p* = 0.0118]. **(H)** Normalizing by the size of each interneuron population (i.e., number of hM_3_D_q_-expressing cells per slice), PV and SST interneurons were associated with similar amounts of post-synaptic inhibition, and both were significantly greater than that associated with VIP interneurons [χ^2^_(2)_ = 20.46, *p* < 0.0001, Kruskal–Wallis test, followed by Dunn’s multiple *post hoc* comparisons; PV vs. SST, *p* > 0.9999; PV vs. VIP, *p* = 0.0064; SST vs. VIP, *p* < 0.0001]. **(I)** Normalizing by the size of each interneuron population, PV interneurons had the greatest effect upon the total ED activity (shown in **Figure 4I**) [*F*_(2,31)_ = 21.03, *p* < 0.0001, one-way ANOVA, followed by *post hoc* Bonferroni’s multiple comparisons tests; PV vs. SST, *p* = 0.0002; PV vs. VIP, *p* < 0.0001; SST vs. VIP, *p* = 0.2808]. ^∗^*p* < 0.05, ^∗∗^*p* < 0.01, ^∗∗∗^*p* < 0.001.

To examine the cellular basis of these effects, we performed voltage clamp recordings to compare how chemogenetic recruitment affects the post-synaptic inhibition converging upon pyramidal neurons. Inhibitory post-synaptic currents were isolated at the reversal potential for glutamatergic current (E_GLUT_) (**Figures 5A,C,E**). For each of the three interneuron populations, CNO-activation of hM_3_D_q_ receptors resulted in a significant increase in post-synaptic inhibitory input (**Figures 5A,C,E**), which was abolished by bath application of tetrodotoxin (1–2 μM; **Figures 5A,C,E**), confirming that it was mediated by action potential-evoked GABA release. Total inhibitory post-synaptic input to pyramidal neurons increased from 11 ± 4.2 pA/ms to 34 ± 6.1 pA/ms when PV neurons were recruited, from 9.3 ± 2.6 pA/ms to 126.6 ± 14.6 pA/ms for SST interneurons and from 5.7 ± 1.1 pA/ms to 16.2 ± 2.4 pA/ms for VIP interneurons (**Figures 5B,D,F**). At the population level, the overall increase in post-synaptic inhibitory input resulting from CNO-mediated activation of hM_3_D_q_ receptors was highest for the SST interneuron population (increase of 116.7 ± 15.5 pA/ms), then the PV interneuron population (increase of 22.6 ± 5.7 pA/ms), and then the VIP interneuron population (increase of 10.3 ± 2.3 pA/ms).

To compare across the different subtypes, we expressed our measurements of inhibitory efficacy in terms of individual interneurons. First, the CNO-induced increase in post-synaptic inhibitory input was normalized by the number of hM_3_D_q_-expressing cells, as determined from stereological cell counts (**Figure 5G**; see “Materials and Methods” section). The increase in inhibitory post-synaptic input was similar for an individual PV interneuron (1.0 ± 0.3 fold, relative to a PV interneuron) and an SST interneuron (1.1 ± 0.1 fold, relative to a PV interneuron), both of which were five times greater than for an individual VIP interneuron (0.2 ± 0.1 fold, relative to a PV interneuron; **Figure 5H**). Second, we normalized the effects upon total ED activity by the number of hM_3_D_q_-expressing cells. Individual PV interneurons were associated with the greatest reduction in epileptiform activity (1.0 ± 0.2 fold, relative to a PV interneuron), which was at least five times more than an individual SST interneuron (0.2 ± 0.04 fold, relative to a PV interneuron) or VIP interneuron (-0.1 ± 0.1 fold, relative to a PV interneuron; **Figure 5I**). These observations are consistent with the idea that, as a result of their peri-somatic targeting and extensive axonal arbors, individual PV interneurons can mediate particularly effective inhibition of pyramidal neurons (Freund and Buzsáki, 1996; Miles et al., 1996).

### Chemogenetic Recruitment of PV Interneurons Attenuates Seizure Activity *in vivo*

**FIGURE 6:**
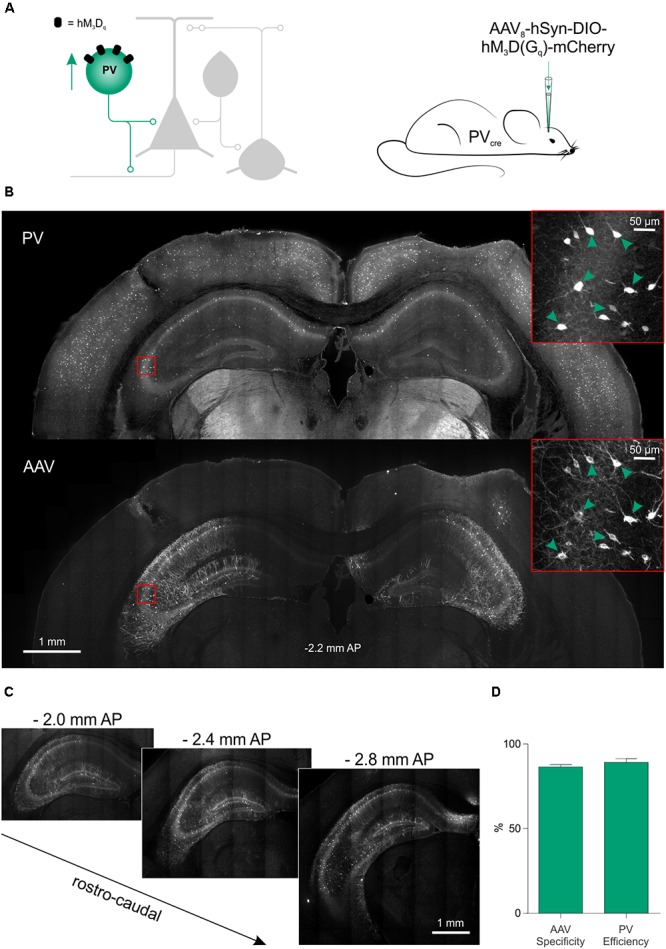
Excitatory DREADDs can be specifically and efficiently targeted to hippocampal PV interneurons *in vivo*. **(A)** To deliver hM_3_D_q_ receptors to PV interneurons *in vivo* (left), PV-cre mice received bilaterally injections of AAV_8_-hSyn-DIO-hM_3_D_q_-mCherry into the ventral and dorsal hippocampus (right). **(B)** Immunohistochemical characterization confirmed that hippocampal PV interneurons (‘PV,’ top) were efficiently transduced with hM_3_D_q_-mCherry (‘AAV,’ bottom). Insets in red squares indicate cells co-expressing PV and hM_3_D_q_-mCherry (arrow heads). **(C)** Serial sections illustrate extensive AAV spread and hM_3_D_q_-mCherry expression throughout the hippocampus. AP, anteroposterior. **(D)** Population data showing high targeting specificity and efficiency of the hM_3_D_q_-mCherry expression in PV interneurons (*N* = 7 animals).

We next assessed the potential of this chemogenetic strategy to reduce seizure activity *in vivo*. Given our findings *in vitro*, the PV interneurons were selected as an effective cell population to target. The hM_3_D_q_ receptor was delivered to PV hippocampal interneurons by bilateral injections of AAV_8_-hSyn-DIO-hM_3_D_q_-mCherry in the hippocampus of 4- to 14-month-old PV-cre mice (**Figure 6A**). The virus was delivered at multiple depths in the ventral and dorsal hippocampus, which resulted in extensive expression of the hM_3_D_q_ receptor across the rostro-caudal axis (**Figures 6B,C**). The majority of all virally transduced neurons were immunopositive for PV (86.42 ± 1.43% specificity) and the majority of PV immunopositive neurons expressed hM_3_D_q_-mCherry (89.17 ± 2.12% efficiency; **Figure 6D**).

**FIGURE 7:**
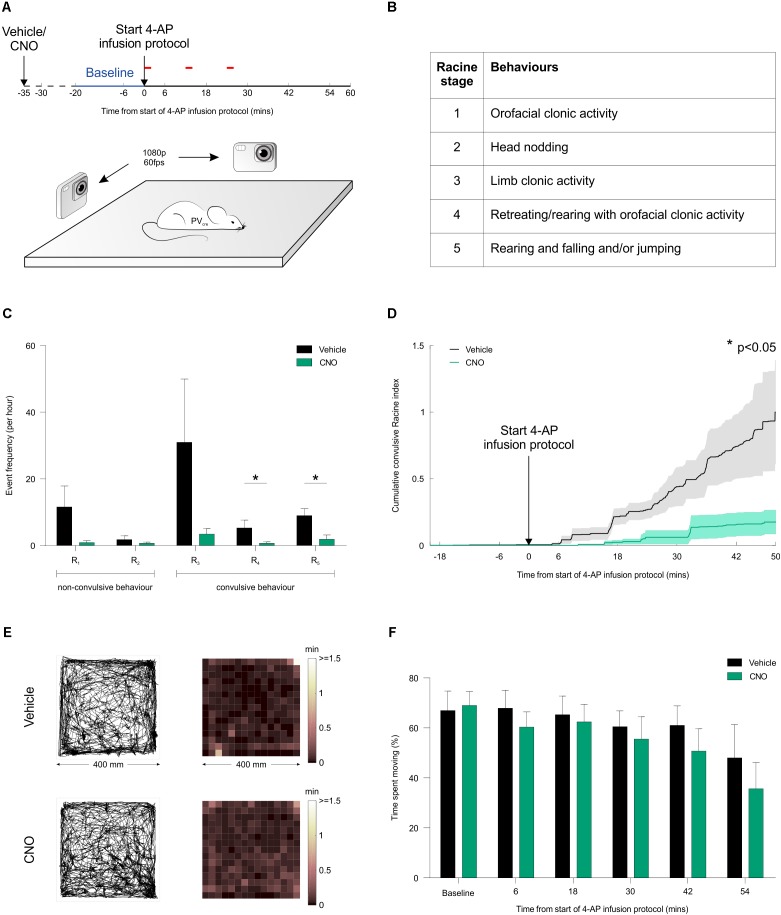
Chemogenetic recruitment of hippocampal PV interneurons suppresses convulsive behaviors *in vivo*. **(A)** Cartoon showing experimental design for assessing seizure activity in animals expressing the hM_3_D_q_ receptor in hippocampal PV interneurons. Fifteen minutes before behavioral monitoring, each animal received an i.p. injection of either vehicle control or CNO. Baseline behavior was then monitored for 20 min (blue period), after which the intra-hippocampal 4-AP infusion protocol was started and monitoring continued for a period of 60 min (red bars indicate the timing of each infusion). Behavior was recorded by two high-speed, high-definition cameras oriented at right angles to one another, while a third camera tracked the animal from above. **(B)** Table describing the Racine scale used to score the animal’s seizure behavior from video analysis offline. **(C)** There was a general tendency for seizure behaviors to be suppressed by CNO across the Racine categories. The frequency of Racine 4 and 5 convulsive events was significantly reduced when CNO was administered compared to vehicle [*N* = 12 vehicle and 10 CNO experiments; Racine 1: *U*_(12,10)_ = 34, *p* = 0.0571; Racine 2: *U*_(12,10)_ = 59.5, *p* > 0.9999; Racine 3: *U*_(12,10)_ = 37, *p* = 0.1335; Racine 4: *U*_(12,10)_ = 31, *p* = 0.0372; Racine 5: *U*_(12,10)_ = 24.5, *p* = 0.0127, two-tailed Mann–Whitney tests]. **(D)** Convulsive behavior, plotted as a normalized cumulative score was significantly lower following administration of CNO compared to vehicle [*N* = 12 vehicle and 10 CNO experiments, *U*_(12,10)_ = 29.5, *p* = 0.0411, two-tailed Mann–Whitney test]. **(E)** For mice expressing the hM_3_D_q_ receptor in hippocampal PV interneurons, plots illustrate tracking data and corresponding spatial distribution of time spent across the behavioral arena (400 mm by 400 mm). Representative data are shown for an animal receiving an i.p. injection of vehicle (top) or CNO (bottom). In each case, data is shown for a 20-min period following the start of the 4-AP infusion protocol. **(F)** There was no difference between the vehicle and CNO groups in terms of the percentage of time spent moving [*N* = 10 vehicle and 11 CNO experiments, treatment: *F*_(1,104)_ = 1.686, *p* = 0.197, two-way ANOVA].

To investigate the effect of activating PV interneurons on seizure activity *in vivo*, acute seizures were triggered by local application of 4-AP to one of the hippocampi, which allows for a controlled focal induction of seizure activity (Salam et al., 2017). Local application of 4-AP likely disrupted the activity of both pyramidal cells and interneurons in a limited brain area and thus generated focal seizures at the injection site, which then spread leading to generalized convulsions. This resembles the clinical situation where focal seizures are frequently initiated in an area of limited abnormal brain tissue from where they spread and propagate through normal brain networks. Such models of acutely triggered focal seizures have been important in the development of anti-epileptic drugs (Kupferberg, 2001) and allow for investigating seizure propagation and spread regardless of the initial cause of seizure initiation. Animals were randomized to receive an i.p. injection of either CNO or vehicle in their first experiment, and then alternated between CNO and vehicle for subsequent experiments. Each i.p. injection was delivered 15 min before the animal was placed in an arena, and their freely moving behavior was monitored for a period of 80 min using high-speed, high-definition cameras (**Figure 7A**). Following a 20-min baseline period, 4-AP was infused directly into the hippocampus according to a spaced delivery protocol (three 4-AP infusions, each separated by 12 min; see “Materials and Methods” section).

To provide a detailed description of seizure activity and seizure spread, each animal’s behavior was scored blindly using the five-point Racine scale (**Figure 7B**), at a sampling frequency of 1 Hz across at least 70 min per experiment (see “Materials and Methods” section). These analyses revealed that CNO-mediated recruitment of hippocampal PV interneurons caused a reduction in the frequency of convulsive behaviors across the Racine 4 and 5 categories (**Figure 7C**). To characterize the temporal nature of these effects, the Racine scoring scale was used to generate an integrated measure of convulsive behavior that could be tracked over time (see “Materials and Methods” section). This integrated measure revealed that CNO-mediated recruitment of hippocampal PV interneurons reduced the occurrence of all convulsive behaviors by more than 80% compared to controls (**Figure 7D**). In contrast, CNO did not have any effect on focal seizure activity (Racine 1-3). Taken together, these results are consistent with the idea that increasing PV activity beyond the area affected by 4-AP reduced seizure spread and propagation. To assess whether this reduction in convulsive seizures was associated with non-specific effects upon behavior, we monitored the animals’ locomotor activity throughout the experiment. The distribution of time spent throughout the arena was indistinguishable between the vehicle and CNO-treated groups (**Figures 7E,F**), supporting the conclusion that the CNO-mediated reduction in convulsive seizures was not associated with a non-specific effect upon locomotor activity. In summary our results suggest that chemogenetic recruitment of PV interneurons is effective at suppressing the spread of convulsive seizure activity *in vivo*.

## Discussion

Here, we use a combination of *in vitro* and *in vivo* studies in rodent models to demonstrate that chemogenetic enhancement of distinct populations of GABAergic interneurons can robustly reduce seizure activity. Targeted whole cell recordings from both pre-synaptic interneurons and post-synaptic principal neurons revealed that PV, SST and VIP interneuron populations all increased their firing rate and synaptic output following CNO-mediated activation of hM_3_D_q_ DREADD receptors. Chemogenetic enhancement of either the PV or SST interneurons decreased drug-resistant synchronized epileptiform activity *in vitro*, through a reduction in the frequency of EDs. In contrast, enhancing VIP interneuron activity did not reduce total epileptiform activity. When accounting for the relative density of cells, PV interneurons generated the strongest effect per cell in terms of their ability to suppress EDs. Finally, to confirm the potential of such an intervention strategy, chemogenetic activation of PV interneurons was shown to produce a fivefold reduction in convulsive behaviors in an *in vivo* model of temporal lobe seizures.

Previous work has shown that targeting pyramidal neurons with inhibitory DREADDs can mitigate seizure activity (Kätzel et al., 2014; Avaliani et al., 2016). The current study extends this by demonstrating that, although they make up a relatively small proportion of the network, chemogenetically recruiting GABAergic populations can mediate robust anti-seizure effects. Enhancing such endogenous inhibitory mechanisms may represent an attractive intervention strategy, as interneurons can exert widespread effects on the tissue and inhibitory circuits are recruited as excitatory network activity intensifies (Trevelyan et al., 2006; Derchansky et al., 2008; Schevon et al., 2012; Cammarota et al., 2013). However, given their diversity, the particular interneuron population that is targeted is likely to be important. In line with this prediction, our data show that chemogenetic enhancement of VIP interneurons did not reduce overall seizure activity, and in fact increased the duration of individual EDs. This is in accordance with observations that the preferential post-synaptic targets of VIP interneurons are other GABAergic interneurons (Acsády et al., 1996), and that activating VIP interneurons may prevent downstream GABAergic interneurons from counteracting seizure-related excitation, since VIP interneurons specialize in disinhibitory control (Lee et al., 2013; Pi et al., 2013). In addition, it is possible that the activation of VIP interneurons leads to direct VIP release, which could increase excitability by enhancing NMDA receptor responses and excitatory transmission (Cunha-Reis et al., 2005; Yang et al., 2009). Consistent with these ideas, optically inhibiting VIP interneuron activity has been shown to generate anti-seizure effects (Khoshkhoo et al., 2017).

In contrast to VIP interneurons, PV and SST interneurons have been shown to preferentially target principal neurons. PV interneurons comprise mainly basket cells and axo-axonic cells, which synapse on the soma and axon-initial segment of pyramidal neurons (Klausberger et al., 2003). The dense axonal branching of PV interneurons is therefore restricted to the pyramidal layer, but exhibits a broad transverse extent (∼1 mm in rodent hippocampus). This can generate widespread synaptic inhibition, with each cell targeting around 1,500–2,000 pyramidal neurons (Freund and Buzsáki, 1996). SST interneurons meanwhile, mainly synapse on the dendrites of pyramidal neurons (Katona et al., 1999), where they regulate dendritic activation (Miles et al., 1996) and may account for as much as half of the firing rate increase following complete removal of inhibition (Lovett-Barron et al., 2012). In addition, SST interneurons can directly release SST when activated, which in turn can reduce neuronal excitability by acting via G-protein coupled receptors (Tallent and Qiu, 2008).

In our experiments, DREADD activation of either PV or SST interneurons resulted in pronounced increases in spike-evoked, post-synaptic inhibitory currents in pyramidal neurons and strong attenuation of spontaneous EDs. For both cell types, we observed a ∼50% reduction in the total ED activity, which was driven by a decrease in the probability of ED initiation. It has been shown that relevant concentrations of CNO can lead to competitive binding of some endogenous receptors (Gomez et al., 2017). However, our control experiments showing that CNO did not affect epileptiform activity in the absence of DREADD expression, support the conclusion that the anti-seizure effects are mediated chemogenetically. At the population level, enhancing SST interneurons generated the largest post-synaptic inhibitory currents in pyramidal neurons. Although when adjusted for cell numbers, we estimated that individual PV interneurons elicited equivalent post-synaptic inhibitory currents and a fivefold greater attenuation of ED activity. These observations are consistent with evidence that GABAergic inputs to the axo-somatic region can exert particularly powerful inhibitory effects (Cobb et al., 1995) and that PV interneurons contribute significantly to a synaptic restraint that can oppose the initiation and propagation of seizure activity (Cammarota et al., 2013; Paz and Huguenard, 2015). In the context of epilepsy, the targeting of PV interneurons may also be more preferable because there are reports that SST interneurons become depleted (Robbins et al., 1991), whereas PV interneurons survive in epileptic animals (Sloviter, 1991; Sloviter et al., 2003) and epilepsy patients (Sloviter et al., 1991).

In agreement with these ideas and our *in vitro* results, we demonstrate that excitatory DREADDs in PV interneurons can generate potent anticonvulsant effects *in vivo*. Direct application of 4-AP to one hippocampus likely generated initial focal seizure activity by blocking K^+^ conductances and altering the activity of both pyramidal and inhibitory neurons within local networks. Under control conditions, these focal seizures spread and progressed to full convulsions (Racine 5). However, chemogenetically increasing the activity of PV interneurons prevented the spread, as reflected by an 80% reduction in convulsive behavior. It has recently been shown that CNO does not cross the blood brain barrier in naive rats, but is rather metabolized to clozapine that can cross the blood brain barrier and activate DREADDs directly (Gomez et al., 2017). It therefore seems likely that the CNO-mediated effects in our *in vivo* studies involved the metabolism of CNO to clozapine, although seizure-related activity could influence the permeability of the blood brain barrier and the relative contribution of CNO. Future studies could test the potential to enhance DREADDs activity in interneurons by direct clozapine administration. Indeed, clozapine is already approved as a drug for use in humans and DREADDs activation requires very low concentrations of clozapine (Gomez et al., 2017), which may make it an attractive option for translational studies.

At the time of submission of our paper, a report was just published that showed that PV interneuron chemogenetic activation in a chronic model of epilepsy was also effective (Wang et al., 2018). The same study shows that chemogenetic inactivation of excitatory neurons resulted in impaired cognitive function. While enhancing PV interneurons may affect cognitive processes (Zou et al., 2016), we observed that reduced convulsive behavior was not associated with general changes in locomotor activity, suggesting that the effects of chemogenetically recruiting PV interneurons may become more evident when the network enters a pre-epileptic state. This indicates that chronic enhancement of interneuron activity would seem to be the more promising strategy for controlling seizure activity compared to direct silencing of excitatory neurons, which might have more serious side effects, e.g., on memory processes. The suppression of focally evoked seizures *in vivo* in the current study complements our results from the *in vitro* drug-resistant model of spontaneous EDs, demonstrating efficacy across both acute and chronic models of seizure activity. More generally, the data confirm that chemogenetically enhancing a specific interneuron population can produce effective suppression of epileptiform synchronization.

A series of studies using rodent models have successfully applied optogenetic approaches for disrupting epileptiform networks. Like chemogenetic approaches, optogenetics lends itself to cell-type targeting and this work has helped define the role of different interneuron types. For example, it has been shown that when combined with real-time seizure detection methods, temporally controlled optogenetic activation of interneuron populations can provide effective disruption of epileptic activity (Krook-Magnuson et al., 2013; Ledri et al., 2014; Sessolo et al., 2015). This work has also revealed that optogenetic activation of interneurons can actually initiate epileptiform activity, in a manner that may depend on the network state (Sessolo et al., 2015; Yekhlef et al., 2015; Assaf and Schiller, 2016; Chang et al., 2018). This phenomenon seems to be associated with the pulsed light-activation and enforced synchronization of interneuron activity, which can then synchronize the network by inducing time-locked post-inhibitory rebound spiking (Sessolo et al., 2015; Chang et al., 2018). In addition to these temporal aspects, optogenetic strategies face other challenges for disrupting seizures, including the delivery of light to structures that may be deep within the brain, or to cells that may be distributed over large regions.

Chemogenetic intervention strategies may mitigate these issues, such as the potential to modulate cellular activity on larger spatial and temporal scales. Effects from chemogenetics can be coordinated across large areas of tissue, due to the systemic delivery of the activating drug (Alexander et al., 2009). Furthermore, the fact that DREADDs are G-protein coupled receptors and act through endogenous cellular mechanisms, may avoid unwanted effects associated with artificial synchronization of the network (Wang et al., 2017). However, their reliance upon the cell’s own intracellular machinery may limit the potential for chemogenetics. Whereas optogenetic strategies have the power to use light energy to move ions against concentration gradients, chemogenetic strategies must rely upon endogenous mechanisms, at least in their current form. Nonetheless, chemogenetics lends itself to translational approaches more readily than optogenetics. We found that activation of G_q_-coupled receptors in PV and SST interneurons can result in sufficient activity of those neurons to dramatically reduce the probability of initiation of epileptic network activity. This has direct implications for drug discovery. As more single-cell RNA sequencing studies are published, the identification of G_q_-coupled receptors that are enriched in PV or SST cells, but not in VIP interneurons and excitatory cells will become feasible, and the present study suggests that those G-protein-coupled receptors represent potential drug targets for temporal lobe epilepsy. Yet, there remain questions about how inert the designed drugs are for the selective activation of the DREADDs, and whether these need to be refined further (Gomez et al., 2017). Any cell-targeted strategy must also consider how the contributions of particular cell types may change in epilepsy. Cell types may be lost or change their signaling at different stages of the disease (Robbins et al., 1991; Cohen et al., 2002; Wang et al., 2017), their contribution may depend on their location relative to the epileptic focus (Sessolo et al., 2015) or their position in the epileptic circuit (Paz and Huguenard, 2015), and their effects may change dynamically during an individual seizure (Ellender et al., 2014). For these reasons, multiple strategies may be required, perhaps using one strategy for pathologically affected cells within the epileptic focus and another strategy for surrounding healthier circuits. Although there are no interneuron subtype-specific promoters yet that would be small enough for AAV-vector-based gene therapy, future progress in this direction would capitalize on the direct translational value of such gene-based strategies, rendering cell-targeted clinical therapies possible.

## Conclusion

The current work supports the use of selective chemogenetic targeting of the inhibitory system as an approach to disrupt epileptiform synchronization. Such a cell-specific pharmacological strategy has the attraction of being controllable and yet avoiding the system-wide effects of drugs that enhance GABA-mediated inhibition in a non-cell-selective fashion.

## Author Contributions

AC, AI, and CA conceived the project and wrote the manuscript. All authors contributed to the design of the experiments and reviewed the manuscript. AC and MS performed the experiments. AC analyzed the data and prepared all figures.

## Conflict of Interest Statement

The authors declare that the research was conducted in the absence of any commercial or financial relationships that could be construed as a potential conflict of interest.
